# Intrinsic Constraints on Sympodial Growth Morphologies of Azooxanthellate Scleractinian Coral *Dendrophyllia*

**DOI:** 10.1371/journal.pone.0063790

**Published:** 2013-05-07

**Authors:** Asuka Sentoku, Yoichi Ezaki

**Affiliations:** Department of Geosciences, Graduate School of Science, Osaka City University, Osaka, Japan; VIB & Katholieke Universiteit Leuven, Belgium

## Abstract

**Background:**

Asexual increase occurs in virtually all colonial organisms. However, little is known about the intrinsic mechanisms that control asexual reproduction and the resultant morphologies of colonies. Scleractinian corals, both symbiotic (zoaxanthellate) and non-symbiotic (azooxanthellate) corals are known to form elaborate colonies. To better understand the growth mechanisms that control species-specific type of colony in azooxanthellate dendrophyllid scleractinian corals, we have studied details of the budding pattern in the sympodial colonies of *Dendrophyllia boschmai* and *Dendrophyllia cribrosa*.

**Principal Findings:**

Budding exhibits the following regularities: (1) the two directive septa of offset corallites are oriented almost perpendicular to the growth direction of parent corallites; (2) offsets generally occur in either of the lateral primary septa that occur on one side of a corallite; the individuals thus show a definite polarity with respect to the directive septa, and only when branching dichotomously offsets occur in both primary septa; (3) the lateral corallites grow more-or-less diagonally upwards; and (4) the regularities and polarities are maintained throughout growth. Given these regularities, *D. boschmai* grows in a zigzag fashion by alternately budding on the right and left sites. In contrast, *D. cribrosa* grows helically by budding at a particular site.

**Conclusions/Significance:**

The strict constraints on budding regularities and shifts in budding sites observed in the sympodial growth forms of corals greatly affect resulting morphologies in azooxanthellate coral colonies. A precise understanding of these intrinsic constraints leads to a fundamental comprehension of colony-forming mechanisms in modular organisms.

## Introduction

The reproduction of colonial organisms occurs both sexually and asexually. Scleractinian corals, especially reef-forming ones, are one of the best known calcifying organisms in the ocean. Most of them reproduce by asexual processes, such as budding or division and form colonies. Many previous studies have focused on the forms of colonies as responses to, and as interrelationships with, external environmental conditions (e.g., [1]–[6]). However, few studies have reported on the specific mechanisms that control astogeny itself and resultant genetically based morphologies (e.g., [7]–[11]). Further investigations are therefore required to specify the means by which asexual reproduction gives rise to offsets and to comprehend how each individual grows and interrelates with others in the context of the whole colony.

The family Dendrophylliidae has a modern worldwide distribution at water depths of 0–2165 m [12]. The family includes both zooxanthellate (e.g., *Turbinaria*) and azooxanthellate (e.g., *Dendrophyllia*) forms, which allows it to exploit a wide range of habitats. According to Cairns [12], the Dendrophylliidae comprises 29 genera and 364 species, of which 20 genera and 166 species are extant. It is the third largest family of the Scleractinia in terms of Holocene species richness (12.6 % of Scleractinia species overall) and the fourth largest in terms of Holocene genus richness (9.0 %). The earliest known fossil record of the Dendrophylliidae is from the Early Cretaceous (Barremian) of Serbia [12].

Although azooxanthellate corals are less influenced by light intensity than are zooxanthellate corals, they show a wide variety of growth forms. Dendroid colonies are roughly divided into monopodial and sympodial forms. In monopodial growth forms, the initial axial corallite of sexual origin prolongs continuously upward from its attachment on the substrate. The axial corallite gives rise to secondary corallites by budding, and the secondary corallites give rise to a sequence of subsequent (lateral) corallites. In contrast, in sympodial growth forms, the initial corallite does not continue to grow; instead, it produces one or two lateral corallites by budding, and the offsets extend the whole colony by both growing and budding.

Sentoku and Ezaki [13]–[15] clarified the regular modes of budding in monopodial dendrophyllid scleractinians, including in *Dendrophyllia arbuscula*, massive *Tubastraea coccinea*, and bushy *Dendrophyllia ehrenbergiana*. Regular budding is defined by budding sites at two or four lateral primary septa, the orientations of directive septa of lateral corallites (almost perpendicular to the growth direction of parent corallites), and the inclination angle of budding (diagonally upward). Importantly, the regularities remain consistent throughout every generation of budding, during the growth of the entire colony. The only differences that occur are related to the locations of budding sites; in *D. ehrenbergiana*, these are limited to two sites on the convex side, revealing a distinct polarity in budding patterns [15]. Subtle modifications of certain parameters, including the budding interval, branch length, corallite size, and inclination angle of lateral corallites, may greatly influence the overall morphology of a colony. However, little is known about the extent to which such intrinsic regularities in budding are applicable to other forms of scleractinians, such as sympodial apart from monopodial scleractinians.

This study examines the regularities in budding of sympodial growth forms of *Dendrophyllia boschmai* and *Dendrophyllia cribrosa* in terms of the sites of budding and their polarities, the orientations of directive septa, and the inclinations of offsets. These two species are suitable as targets for investigating budding processes because they possess comparatively large calices and carved corallites parallel to the directive septa. By analyzing these species, it is possible to comprehensively understand the nature of intrinsic constraints on budding and on resultant colonial forms. Additionally, it is possible to grasp how a variety of colonial growth forms are generated through a series of specific budding processes. Most importantly, the findings, which are largely dependent upon skeletal data on the formation of modules and modes of assembly, lead to an appreciation of other colonial growth forms with or without skeletons, both extant and extinct.

## Materials and Methods

**Figure 1:**
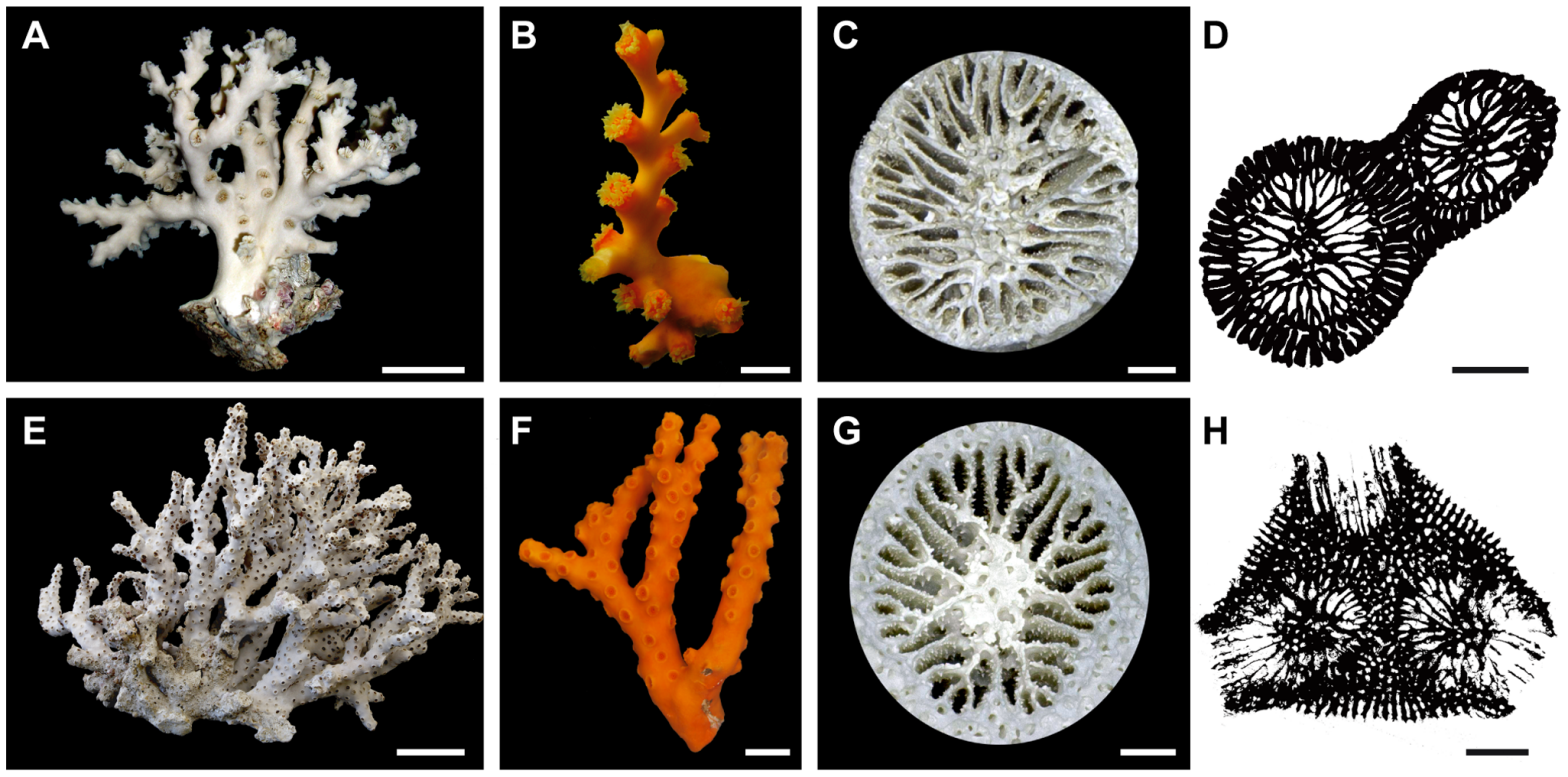
*Dendrophyllia boschmai* and *Dendrophyllia cribrosa*. A–D, *Dendrophyllia boschmai* (OCU 6652–6661). E–H, *Dendrophyllia cribrosa* (OCU 6662–6671). A, E, Side views of whole colonies. Scale bars = 50 mm. B, F, Living colonies surrounded by orange-colored coenosteum tissues. Scale bars = 10 mm. C, G, Calicular view showing a Pourtalès septal plan. Scale bars = 1 mm. D, H, Drawings of transverse thin sections. Note that individual corallites are connected with coenosteum skeletons. Scale bars = 3 mm.

**Figure 2:**
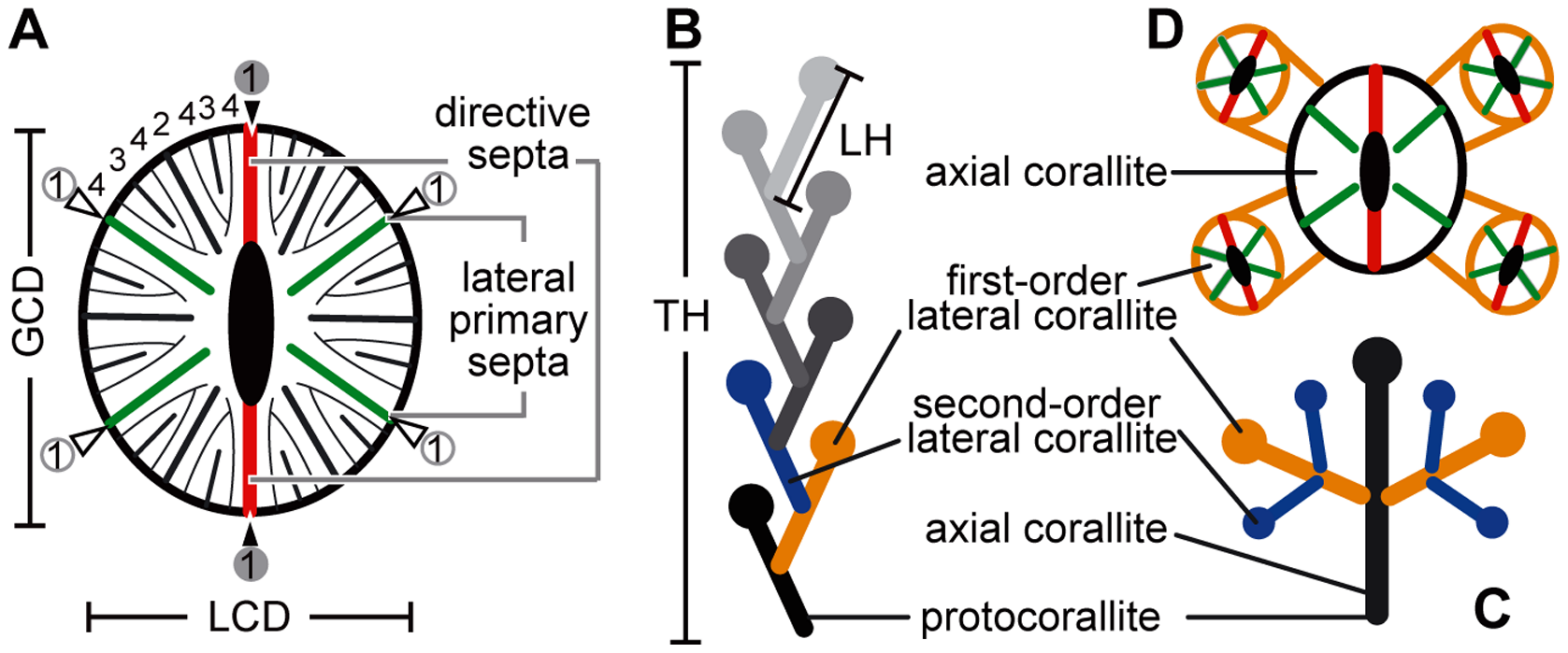
Schematic constructions of calicular features, and sympodial as well as monopodial forms of colonies. A, Calicular view, showing the two opposite directive septa, the greater calicular diameter (GCD), and the lesser calicular diameter (LCD). Numbers indicate the cycles of septa; the first cycle of septa is circled. The two directive septa are indicated by black triangles, whereas the four remaining septa are shown by white triangles. B, Sympodial form of colony. TH indicates the corallum height, and LH the lateral corallum length. Note that the colony does not possess an axial corallite. C, Monopodial form of colony. D, Budding site of monopodial colony form, where offsets generally occur in some of the lateral primary septa.

We examined 45 coralla of *Dendrophyllia boschmai* (Fig. 1A–D) and 43 coralla of *D. cribrosa* (Fig. 1E–H), collected at water depths of 7–165 m offshore of Minabe (Wakayama Prefecture), Amakusa (Nagasaki Prefecture), and Minamiise (Mie Prefecture) in southwest Japan. Of these, 20 coralla were selected for analysis and the morphometric parameters of constituent individuals were measured. The greater calicular diameter (GCD; *sensu* [16]) is oriented parallel to the two directive septa (Fig. 2A). The maximum GCD of *D. boschmai* is 11.2 mm and the maximum lesser calicular diameter (LCD; *sensu* [16]) is 9.3 mm (Fig. 2A). The maximum GCD of *D. cribrosa* is 6.0 mm and the maximum LCD is 5.1 mm. The studied specimens are registered in the Department of Geosciences, Graduate School of Science, Osaka City University, Japan (OCU 6669–6688).

**Figure 3:**
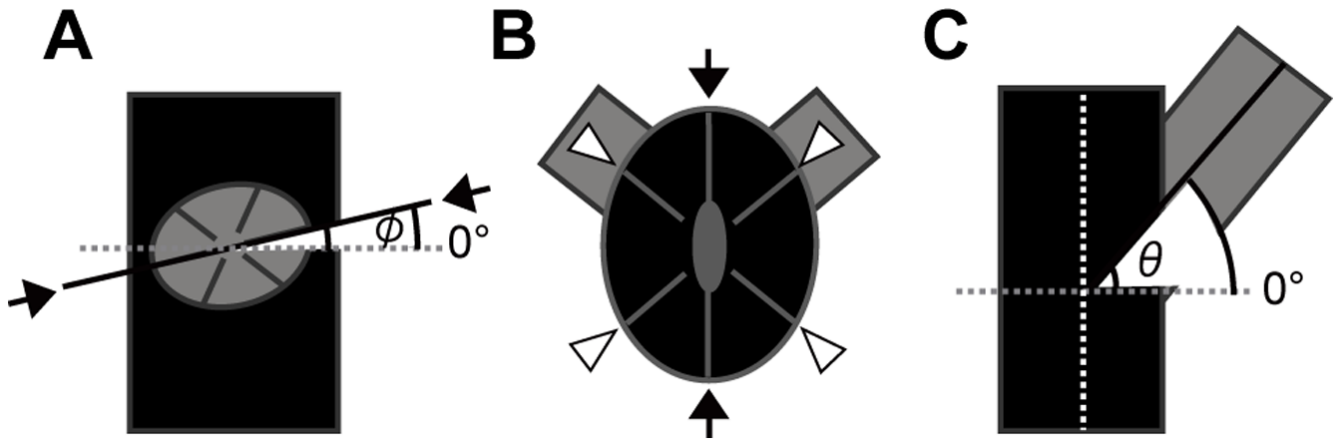
Measured parts of the lateral corallites of a colony. A, Orientation of the directive septa, defined as the angle (φ) between a line connecting the two directive septa (solid line) and a horizontal line (dotted). B, Budding sites. The two directive septa are indicated by arrows, and the four lateral primary septa are indicated by white triangles. C, Inclination of budding, defined as the angle between the direction of the lateral corallites (solid line) and a horizontal line (dotted line) oriented perpendicular to the growth direction of the parent corallite (white dotted line).

**Figure 4:**
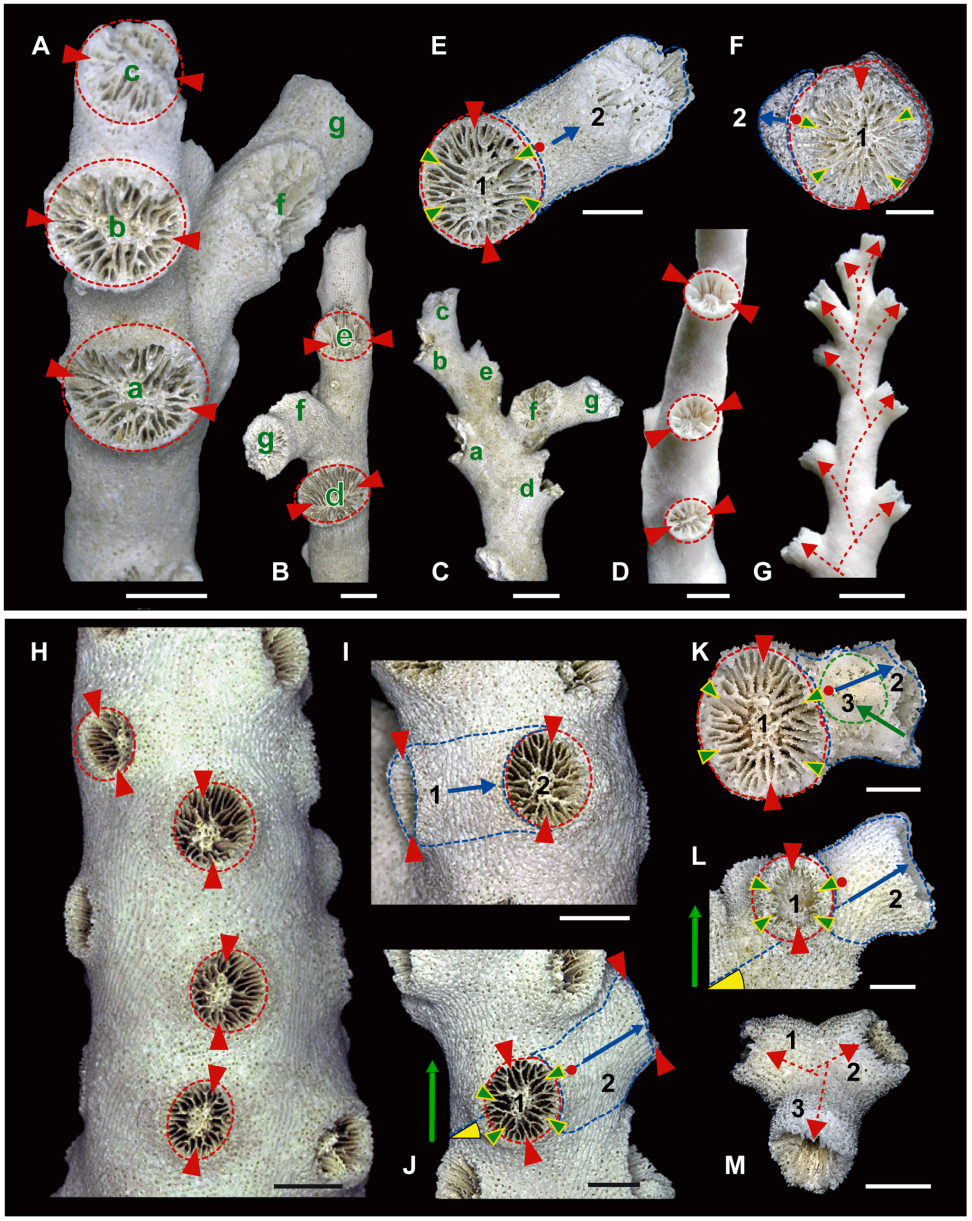
Results of regularities in budding (red triangles, directive septa; green triangles, the four lateral primary septa; red circles, budding site; 1, parent corallites; 2, daughter corallites; 3, grandchild corallites; arrows, growth direction). A–F, *Dendrophyllia boschmai* (OCU 6654–6658). G–L, *Dendrophyllia cribrosa* (OCU 6664–6668). A–D and H–J, Orientations of directive septa. A–D, The directive septa of corallites, which are apparently perpendicular to the growth direction of the branch. Scale bars = 10 mm. A, Left-side view of the colony shown in C. B, Right-side view of the colony shown in C. C, Front view of the colony shown in A and B. D, Right-side view of the colony shown in G. H, The directive septa of corallites, which are apparently parallel to the growth direction of the branch. Scale bars = 5 mm. I, J, Actual relationships between directive septa of parent and its derived corallites. Scale bars = 5 mm. E, F, J, and L, Budding sites. Scale bars = 5 mm. The offsets ordinarily occur in either of the primary septum on one side of individual corallites, excluding the directive septum. G and M, Inclinations of budding. Scale bars = 10 mm.

To assess regularities in budding, we measured the following features: (1) GCD, (2) LCD, (3) length of lateral corallites (lateral height, LH; Fig. 2B), and (4) height of the whole coralla (total height, TH; Fig. 2B). For the largest known colony of *D. boschmai*, the TH is 220.4 mm and the LH is 21.0 mm; the colony consist of approximately 300 individuals. For the largest known colony, of *D. cribrosa*, the TH is 312.6 mm and the LH is 18.3 mm; the colony consist of approximately 600 individuals. We photographed relevant coralla at various angles and magnifications to determine: (1) the budding sites, (2) the orientations of directive septa, and (3) the inclinations of lateral corallites. When necessary, measurements were obtained using image-processing software (Adobe Photoshop) and an electronic caliper. The definitions of the measured features are as follows:

1. Orientations of directive septa: the angle between the directive septa of a lateral corallite and the growth direction of the parent corallite (Fig. 3A).
2. Budding sites: the sites of budding on parent septa, around which the offsets appear (Fig. 3B).
3. Inclination of budding: the inclination angle of lateral corallites, measured as the deviation from perpendicular to the growth direction of the parent corallite (Fig. 3C).

## Results

### Orientations of Directive Septa

**Figure 5:**
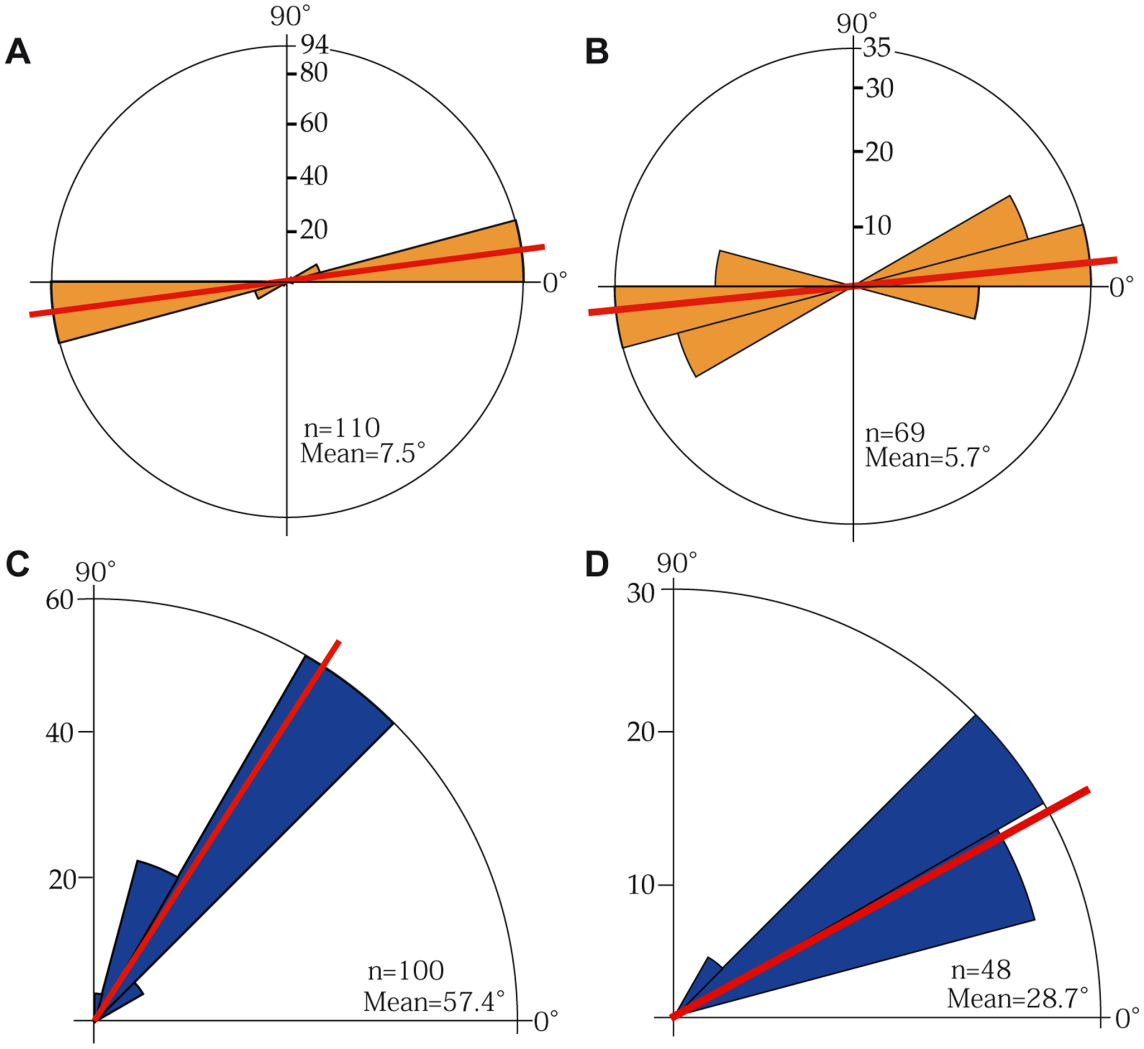
Rose diagrams showing the divergent budding angles between lateral corallites, their orientations and the inclination of the lateral corallites of *Dendrophyllia boschmai* (A, C) and *Dendrophyllia cribrosa* (B, D). A–B, Orientations of directive septa. C, Inclinations of lateral corallites.

Figure 4A–D and 5A show that the orientations of the directive septa of corallites in *Dendrophyllia boschmai* are nearly perpendicular to the growth directions of branches. For 94 of the 110 corallites (85 %), the orientations fall within the range 0°–10° (average, 7.5°). In contrast, Figure 4H–J and 5B show that the orientations of the directive septa of corallites of *D. cribrosa* are apparently parallel to the growth direction of the branch (Fig. 4H). However, careful observations of the relationships between them (red triangles, Fig. 4I, J) indicate that the orientations are clearly perpendicular to the growth direction of the parent corallites (blue arrows, Fig. 4I, J). For 35 of the 69 corallites (50.7 %), the orientations fall within the range 0°–10° (average, 5.7°). The directive septa of lateral corallites are thus commonly oriented nearly perpendicular to those of the immediate parent corallites.

### Budding Sites

Analyses of budding sites using a series of transverse sections and unbroken observations of 105 derived corallites in *D. boschmai* reveal that the offsets arise at a specific cycle of septal formation of the parent corallites (Figs. 1D–F). The offsets always occur near the lateral primary septum on one side only (red circles, Fig. 4E, F). Similarly, analyses of the budding sites of 62 lateral corallites in *D. cribrosa* also reveal that offsets arise at a specific cycle of septal formation (Fig. 4J–L). As in *D. boschmai*, the offsets always occur near the lateral primary septum on one side only (red circles, Fig. 4J–L).

Irrespective of the budding generation, the lateral corallites never bud from two opposite directive septa, but only at one specific primary lateral septum on a particular side of a colony.

### Inclination of Budding

Figure 4G and 5C show the inclination angle of budding in *D. boschmai*. Out of 100 corallites, 60 (60 %) show inclination angles in the range of 45°–60° (average, 57.4°), which represents diagonally upward growth of lateral corallites.

Similarly, Fig. 4M shows the inclination angles of budding in *D. cribrosa*. Out of 48 coralites, 30 (62 %) show inclination angles in the range of 30° to 45° (average, 28.7°).

Inclination of budding in *D*. *cribrosa* shows the acute angles at the base of budding sites and becomes obtuse by curving the growth direction of individual corallites convex upward. A successive budding forms a spiral structure in opposite right- and left-handed directions. The green arrows in Fig. 4J and L show the growth direction of the spiral axis of the colony, and the blue arrows show the growth direction of the individual budding corallites. Importantly, the budding corallites grow diagonally upward in the growth direction of the colony (Fig. 4J, L).

### Budding Sites and Growth Modes

**Figure 6:**
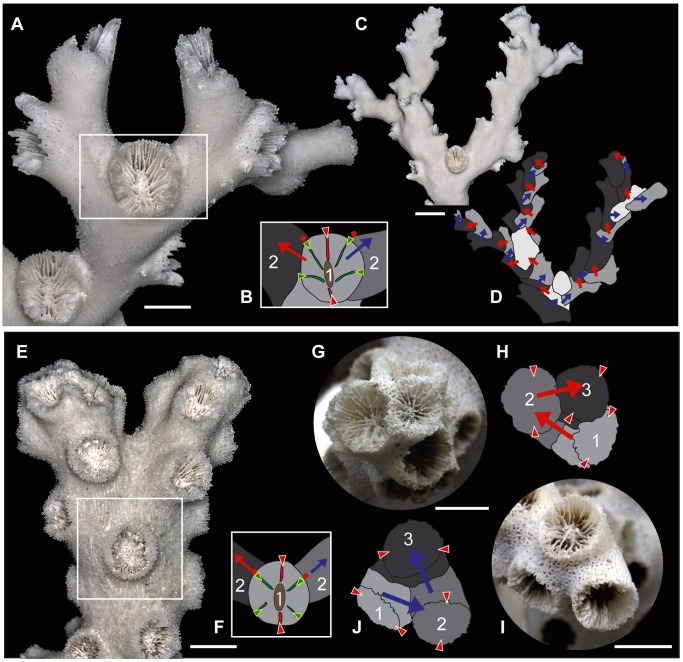
Branching area and growth direction (red triangles, directive septa; green triangles, the four lateral primary septa; red circles, budding site; arrows, growth direction; 1, parent corallites; 2, daughter corallites; 3, grandchild corallites). A–D, *Dendrophyllia boschmai* (OCU 6661). E–I, *Dendrophyllia cribrosa* (OCU 6671). Scale bars = 5 mm. A and E, Branching area of colony. B and F, Drawings of A and E, showing the branching area and budding sites. C, Front view. D, Drawing of C, showing the branching area and growth direction. G, Top view of the colony shown in the left side of E. H, A drawing of G, showing the sinistral growth direction. I, Top view of the colony shown in the right side of E. J, A drawing of I, showing the dextral growth direction.

Figure 6A–D shows the budding sites and growth modes of *D. boschmai*. Ordinarily, *D. boschma*i buds alternately at one of the two lateral primary septa of a corallite (Figs. 4E, F, 6D). Therefore, the colony grows upwards in a zigzag fashion (Figs. 4G, 6C–D) without showing an axial corallite in a sympodial apart from monopodial form. However, when branching dichotomously, offsets occur in both of the two lateral primary septa (Fig. 6A–B), in which case the colony is bilaterally symmetrical along the directive septa of a branching corallite, with a definite polarity. After branching, individuals again grow upwards in a zigzag fashion.

**Figure 7:**
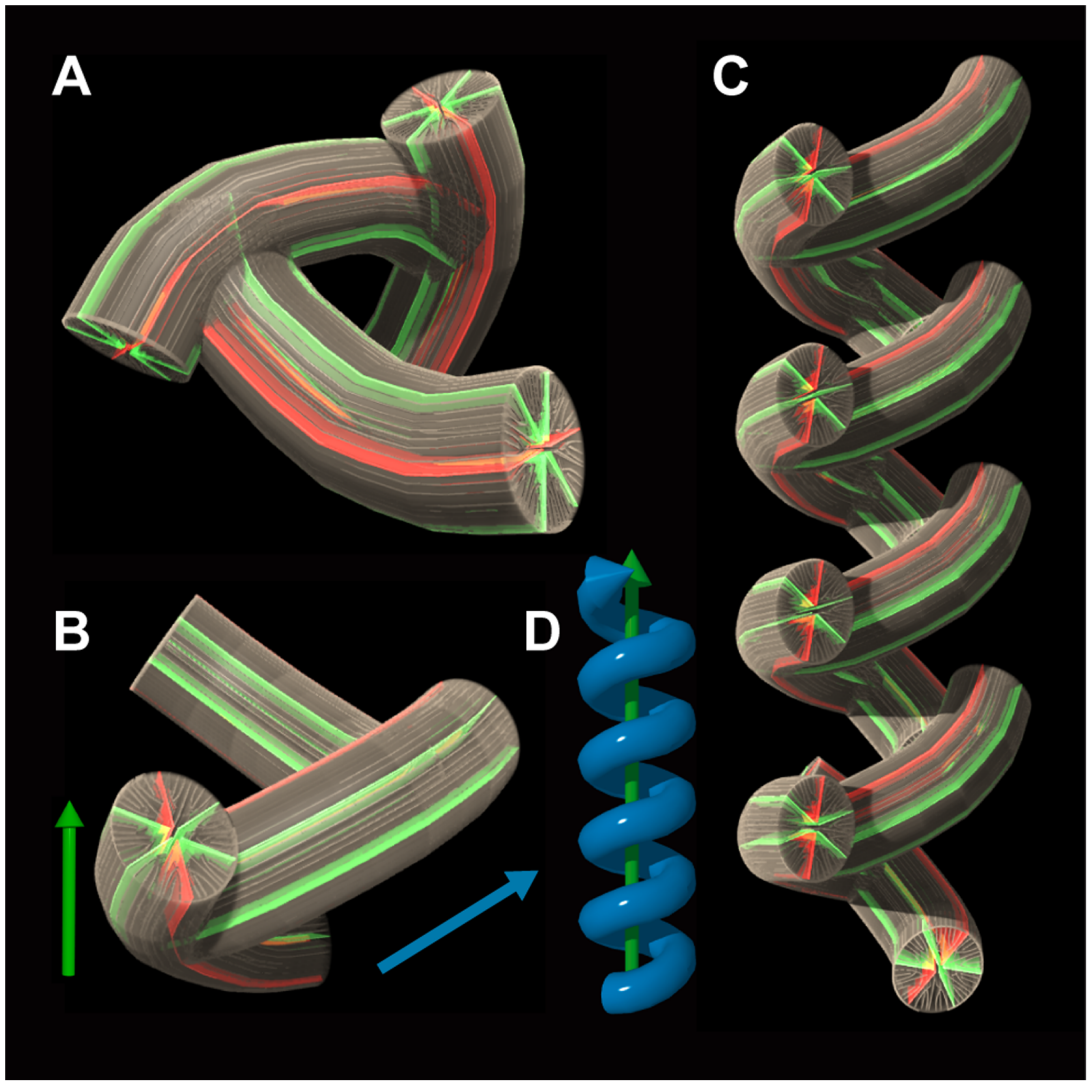
Schematic diagrams of spiral architecture in ***Dendrophyllia cribrosa*** **(green arrows, growth direction of the colony; blue arrow, growth direction of a budding corallite).** A, One round of spiral architecture, which is made up of three individuals. The angle between a parent and its daughter corallites is approximately 120°. B, Growth directions of the individuals. C, Schematic view of the internal structure in *D. cribrosa.* D, Growth direction of the colony.

Figure 6E and F shows the budding sites and growth modes of *D. cribrosa*. Normally, *D*. *cribrosa* consistently buds at a specific site at the two lateral primary septa of a corallite (Figs. 4K, L, 6G–J), and grows helically upwards (Figs. 6G–J, 7). Individual corallites are eventually immersed with coenosteum skeletons, without showing an axial corallite (Figs. 1H, 7C) in a sympodial apart from monopodial form. However, when branching dichotomously, offsets occur in both of the two lateral primary septa (Fig. 6E, F), in which case the colony is bilaterally symmetry along the directive septa of the branching corallite, with a definite polarity. Figure 6G–J shows top views of a colony. After branching, individuals again grow upwards in a helical fashion, with each branch rotating in opposite right- and left-handed directions (Figs. 6H, J, 7A–D).

## Discussion

### Regularities in Budding

**Figure 8:**
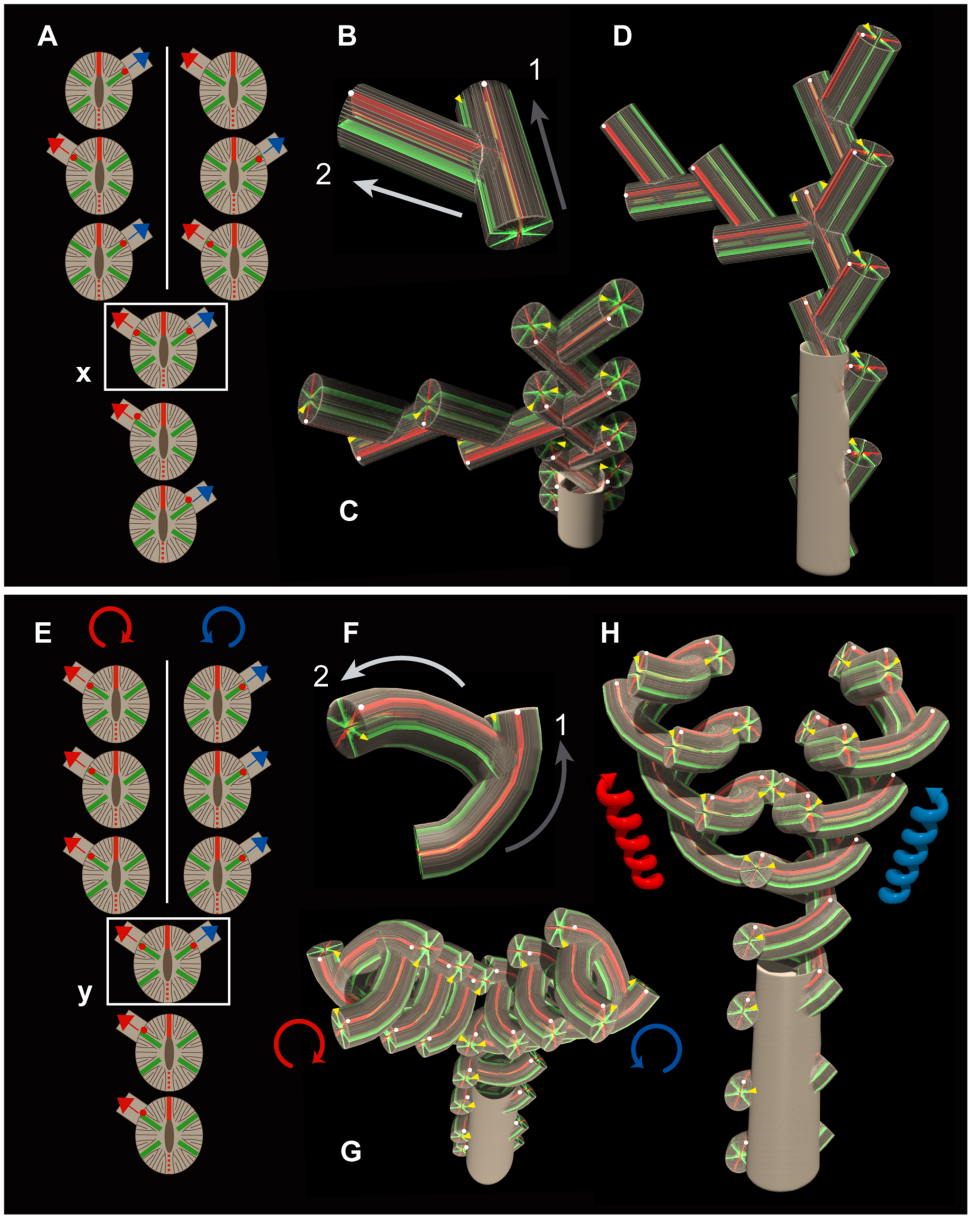
Schematic diagrams of the budding modes and the polarity (red bars, directive septa; green bars, the four lateral primary septa; red circles, budding site; white circles, polarity; arrows, growth direction; white lines, plane of bilateral symmetry). A–D, *Dendrophyllia boschmai.* E–H, *Dendrophyllia cribrosa*. A and E, Locations of budding sites and growth direction of individual corallites. x and y, dichotomous branching areas of a colony. B and F, Essential units of sympodial growth. C and G, Top views. D and H, Lateral views. Notably, given these regularities, *D. boschmai* grows in a zigzag fashion by budding at right and left sites alternatively, whereas *D. cribrosa* grows helically by budding at a particular site. In addition, *D. cribrosa* inevitably changes the direction of rotation in right and left branches after branching due to the presence of developmental constraints for maintaining polarity.

Figure 8 provides schematic views of the sympodial forms of *Dendrophyllia boschmai* and *D*. *cribrosa.* Four regularities are common to both species: (1) the directive septa of each corallite are oriented almost perpendicular to the growth directions of parent septa (Figs. 4A–D, H–J, 8B, F); (2) the lateral corallites never occur in the sectors of the two directive septa, but at specific primary septa on a particular side of the colony (Figs. 4E–F, J–L, 8A, E). Offsets occur in the two lateral primary septa only when branching dichotomously (Figs. 6A–B, E–F, 8A, E); (3) the individual budding corallites grow acute (diagonally upwards) at least in the beginning with respect to the growth direction of immediately preceding corallites (Fig. 8B, F); (4) irrespective of generation of budding, those regularities remain consistent during the growth of the entire colony (Fig. 8D, H).

The differences between the sympodial colony forms of *D. boschmai* and *D*. *cribrosa* are generated by the following factors: (1) budding offsets of *D. boschmai* grow upwards, whereas those of *D*. *cribrosa* finally grow downwards with respect to the growth direction of immediately preceding corallites (Fig. 8B, F); when the angle between the parent and daughter corallite is obtuse, then one round of the spiral structure of the branch is composed of fewer individuals. In the case of *D. cribrosa*, one spiral structure is constructed of effectively only three individuals (i.e., the angle between the parent and its daughter corallites is approximately 120°; Fig. 7A), although offset corallites invariably grow upward with respect to the growth direction of their branches (Fig. 7B–D); and (2) regarding the budding sites on one side of corallites, offset corallites of *D. boschmai* always occur alternately in either of the lateral primary septa, whereas those of *D. cribrosa* normally occur at a single specific site (Fig. 8A, E).

It is considered that budding of lateral corallites in various directions is advantageous, as this strategy makes full use of available vacant space and maximizes the number of individuals. It has been pointed out that the budding sites in a monopodial growth form are located at four lateral primary septa (e.g., in *D. arbuscula* and *T. coccinea* [13]–[14]) or the two primary septa on one side of the corallites (e.g., *D. ehrenbergiana* [15]). In contrast, in both of the sympodial forms studied herein, the budding sites are normally restricted to one lateral primary septum. The two directive sectors do not give rise to offsets as in other azooxanthellate scleractinians (e.g., as in the monopodial form of *Dendrophyllia* and the sympodial form of oculinid *Cyathelia axillari*s; [15], [17]), implying the presence of strict developmental constraints on budding. In the Scleractinia, the six primary septa are formed first, thus regulating ensuing skeletogenesis by the determination of sites of septal insertion, and budding of invaluable growth axes, thereby controlling the overall growth form of the colony.

### Polarity of Individual Corallites and Resultant Colonial Growth

Sentoku and Ezaki [15] clarified that the Scleractinia exhibit a distinct polarity, as is clearly evident in the budding sites of *D. ehrenbergiana*, although the group shows an apparently radial symmetry in septal arrangement. Clear axial corallites are present in *D. ehrenbergiana*, and individual corallites are curved parallel to their directive septa. The lateral corallites uniquely occur at the two lateral primary septa on a convex side, showing a plane of bilateral symmetry with a distinct polarity. In contrast, polarities in the sympodial growth forms of coralla considered herein (i.e., in *D. boschmai* and *D. cribrosa*) are unclear, because individual corallites exhibit neither curvature in growth nor axial corallites. However, a careful examination of the budding sites in the two species indicates that offsets invariably occur at specific septa on a particular side of corallites, irrespective of generation (Fig. 8A, E).

When branching dichotomously, offsets always occur simultaneously at the two lateral primary septa of corallites, and possess a definite polarity, emphasizing a bilateral symmetry along the directive septa which possibly corresponds to the bilateral dorsal-ventral axes of the corallites (Fig. 8A–x, E–y). Remarkably, in one case, the coiling direction of *D. cribrosa* is completely opposite (dextral vs. sinistral) in adjacent branches (Fig. 6H, J); this is due to the maintenance of a specific polarity throughout successive offsetting (Fig. 8F–H). It appears that individuals always give rise to offsets at specific primary lateral septa within two possible sites, which are used only once when branching. Such underlying regularities in budding, especially the presence of budding polarities, eventually have a strong effect on the overall form of colonies. Similarly, developmental constraints on the maintenance of polarity have been observed in the case of rugosan division [18].

### Ecological Significance of Different Types of Sympodial Growth Forms

The calicular diameter of *D. cribrosa* (GCD = 5 mm) is smaller than that of *D. boschmai* (GCD = 10 mm). However, the colony size of *D. cribrosa* (TH = 40 cm) is larger than that of *D. boschmai* (TH = 20 cm). As compared to the zigzag growth form of *D. boschmai*, *D. cribrosa* develops stout branches by secreting coenosteum skeletons over the internal spiral-forming individuals. Extant dendrophylliids occur worldwide (except off Antarctica), and live at depths from the intertidal to 2165 m [12]. The constituent genera exhibit a variety of solitary and colonial growth forms, allowing them to exploit a wide range of ecological niches [12]. Colonies of *Dendrophyllia* form a variety of growth forms while maintaining specific regularities in budding. Even in the case of the same sympodial growth species, *D. boschmai* (40–165 m [12]) is totally different from *D. cribrosa* (7–40 m [12]) in habitat depth. It is highly probable that these differences result from habitat segregation, depending largely on the physical properties of sea water such as wave and current intensities.

In the case of an average-sized monopodial *Dendrophyllia arbuscula* (TH = ∼200 mm, LH = 30 mm, GCD = 10 mm), there are approximately 35 corallites and up to four generations of individuals in the colony. In contrast, the maximum LH of sympodial forms is generally below 10 cm, which is far shorter than what is observed in monopodial forms. Individual branches are short, and the vacant space available for further growth and budding regulates more or less the overall growth morphology of colonies. In contrast, thorough examination of a number of dendrophyllid species reveals that the overall size of sympodial colonies tends to be larger than that of monopodial colonies typical of *D. arbuscula.* If the budding interval necessary for further growth is shorter, the efficiency of increasing number of corallites is far greater. Under the circumstances, the sympodial morphology in some cases leads to more effective utilization of resources, in light of growth space and food intake availability. Throughout the growth of the colony, regularities in the pattern and frequency of budding are genetically predetermined, thus limiting the construction of colonial architectures. Such growth restrictions enable the formation of large-sized colonies with large numbers of constituent corallites under only a few rules of budding to maximum use of available space and intake of nutrients. Strict developmental constraints, along with subtle modifications in related parameters, thus greatly affect the colonial growth of sympodial, rather than monopodial, forms of colonies. The types of budding in sympodial forms are effective and adaptive in the case of limited resources (e.g., growth space and nutrient uptake availability). However, sympodial forms seem to grow more slowly than monopodial forms, and require more abundant foods. For these reasons, the habitable and distributional ranges of sympodial forms of colonies are so restricted. Regardless, each sympodial species is expected to adopt the colony-forming strategies of their own suitable for ambient habitat conditions, even under the strict developmental constraints on budding.
